# Factors associated with unfavourable treatment outcomes among tuberculosis patients at health facilities of Maseru, Lesotho

**DOI:** 10.4102/safp.v66i1.6004

**Published:** 2024-10-21

**Authors:** Masechaba M. Leketa, Slindile Zondi, Lindiwe Cele, Mmampedi Mathibe, Phuti Ngwepe

**Affiliations:** 1 Department of Public Health, Faculty of Health Sciences, Sefako Makgatho Health Sciences University, Pretoria, South Africa; 2 Department of Statistical Sciences, Faculty of Health Science, University of Cape Town, Cape Town, South Africa

**Keywords:** tuberculosis, treatment success rate, Maseru, unsuccessful TB treatment, mycobacterium tuberculosis

## Abstract

Tuberculosis (TB) treatment success rates (TSR) remain low in Lesotho. The country achieved TSR of 76% in 2020, which is far below the World Health Organization’s 95% target set for 2030. This study determined the level of treatment success and factors associated with unfavourable treatment outcomes. This descriptive cross-sectional study used patient records to perform descriptive and logistic regression analyses. Sixty-one per cent of the 336 TB patients were male. Two hundred and eighty-nine had favourable outcomes, of which 10% died in the course of treatment. Patients ≤ 44 years old, and those with supported treatment, had lower odds of unfavourable treatment outcomes, respectively, adjusted odds ration [aOR] = 0.39 (95% CI: 0.19–0.78) and aOR = 0.22 (95% CI: 0.08–0.62), *p* < 0.05.

**Contribution:**

The death of patients while on TB treatment needs to be addressed, including heightened advocacy for supported treatment.

## Introduction

Tuberculosis (TB) remains a significant global health challenge, despite being preventable and treatable, affecting approximately one fourth of the global population. The African region is reported to contribute the greatest number of TB infections, with 24 out of 30 countries with the highest TB burden located in this region; approximately 2.5 million cases are said to have occurred in the sub-Saharan Africa (SSA), in 2017. Lesotho had one of the highest TB incidence rates in the world, with an estimated 614 cases per 100 000 populations, in 2021. Lesotho is also among the countries with the highest prevalence of multidrug-resistant tuberculosis (MDRTB) in the SSA. The drivers of the TB disease in the country include the high prevalence of the human immunodeficiency virus (HIV) of 19.3% among the people aged between 15- and 49-year old population in 2022, with 59% of persons with TB in Lesotho, co-infected with HIV.^1,2,3^

The standard TB treatment regimen involves an intensive 2-month phase utilising four medications to eradicate Mycobacterium tuberculosis (MTB), followed by a continuation phase lasting up to 4 months with two medications to prevent relapse. Despite these treatment protocols, achieving successful outcomes remains a challenge especially for countries in Africa including Lesotho, which achieved a treatment success rate (TSR) of 76% in 2020, falling short of the World Health Organization (WHO) TSR target.^1,4,5,6^ Various interventions, including TB preventive treatment, vaccination with the Bacillus Calmette-Guerin (BCG) vaccine and targeted medication for high-risk groups, aim to reduce TB progression to active disease. However, challenges such as socioeconomic factors, comorbidities such as HIV and healthcare system deficiencies hinder effective TB management. Meanwhile, efforts to ensure successful TB therapy outcomes, such as through the directly observed treatment short-course (DOTS) programme, are essential for improving treatment success. Nevertheless, factors such as drug resistance and poor support for adherence contribute to unfavourable treatment outcomes.^7,8,9,10^

The United Nation’s (UN’s) End TB Strategy and the Sustainable Development Goals (SDGs) aim to significantly reduce TB fatalities, new cases and associated costs. However, the coronavirus disease 2019 (COVID-19) pandemic has disrupted TB control efforts, impacting progress towards these goals. Nonetheless, there has been a global decrease in newly diagnosed TB cases from 7.1 million in 2019 to 5.8 million in 2020 although TB deaths have increased from 1.2 million in 2019 to 1.3 million in 2020 because of limited access to diagnosis and treatment. Lesotho, recognising the urgency of TB control, has adopted the enhanced TB diagnostics and introduced the fixed drug combinations (FDCs) since 2009 to improve treatment adherence. Despite these efforts, Lesotho remains among the countries with high TB infections, underscoring the need for further research to identify factors contributing to unfavourable TB treatment outcomes and propose effective strategies to address them.^11,12,13^

Hence, this study aimed to determine the factors influencing unfavourable TB treatment outcomes and suggest areas for interventions to improve TSRs, particularly within the Christian Health Association of Lesotho (CHAL) health facilities in the Maseru District; additionally, this research seeks to contribute to the global efforts to combat TB and support Lesotho’s aspirations to achieve SDG-related targets by 2030. The WHO defines treatment outcomes of cured and treatment completed as successful treatment outcomes, while outcomes ‘defaulted’, ‘died’, ‘transferred out’ and ‘treatment failure’ are categorised as unsuccessful treatment outcomes.^4^

## Methods

### Study design

This descriptive cross-sectional and quantitative study conducted a retrospective review of medical records of TB patients that had been initiated on anti-TB treatment between 2018 and 2021 at selected health facilities located in the district of Maseru in Lesotho

### Study setting and study population

The study setting was seven health facilities that are church-owned and operated by the CHAL within the Maseru District. Maseru is the capital city of Lesotho, and the largest of the 10 districts of Lesotho. Maseru is located on the Caledon River, which forms the west-central border between Lesotho and South Africa and is home to a population of 519 186 people. Gender distribution indicates that there are more females than males, 269 327 versus 249 859, with the majority of the population in the younger age groups 0–39 years.^14^ Disease profiling indicates that Lesotho has the second highest TB incidence in the world, estimated at 724 cases per 100 000 populations, of which 73% are co-infected with HIV.^15,16^

Christian Health Association of Lesotho is a voluntary organisation thatcomprises six member churches, namely Roman Catholic, Assemblies of God, Seventh day Adventist Church of Southern Africa, Lesotho Evangelical Church in Southern Africa, Church of the Bible Covenant and the Anglican church in Lesotho. The services offered at all the selected seven facilities include among others, TB and HIV, outpatient, maternal health services, antenatal care, labour and delivery, under five clinics, cervical cancer screening and health education. The organisation serves 8 hospitals, 71 health centres across Lesotho and 4 nursing schools, where it provides 40% of healthcare in the country.^17^ Five of the selected seven health facilities are in the urban side of Maseru, and they serve both poor and able communities as they are closer to town and the community is mostly literate. The remaining two rural facilities serve mostly poor communities with lower literacy levels.

### Sample size and sampling technique

The sample size was determined using the Raosoft sample size calculator, which is available online.^18^ The researchers considered the 1470 total number of TB patients who were initiated on anti-TB treatment between 2018 and 2021 for the population size. A confidence level of 95% was chosen, which corresponds to a 5% margin of error, and the response distribution was set at 50%. After adding a 10% buffer, the final sample size was 336.

The seven health facilities were selected based on headcount and ease of access. These were treated as strata and the number of patient files required to be selected from each of the facilities were determined in proportion to the population size. For the patient file selection, the study used the simple random sampling strategy (SRS) by assigning numbers to the patients’ files before using the online random number generator until the required number of files from each facility was selected.

### Definition of variables

The primary aim of this study was to measure the rate of treatment success and investigate the factors associated with unfavourable treatment outcomes. Treatment success was defined based on whether the patient was categorised as cured or treatment completed. For the factors associated with unfavourable treatment outcome, treatment categories of treatment failure, treatment defaulted and lost to follow-up were categorised as unfavourable treatment outcome ‘yes’, while those who were cured and treatment completed were categorised as unfavourable treatment outcome, ‘no’. The TSR was calculated by adding the total number of patients who were cured and those who completed treatment and this was divided by the total number of patients who were enrolled in the study cohort multiplied by hundred. Patients who died during the course of treatment were excluded from further analysis. The sociodemographic and clinical data were used as independent variables.

### Data entry and analysis

The researchers conducted a retrospective review of patients’ records using the data collection tool developed by the researchers as per the data elements from the Lesotho National TB Register and TB Card. The collected data included the sociodemographic data (age, sex, employment status, etc.), and the clinical history, such as the history of previous TB treatment, having a treatment supporter, substance use, comorbidities and being a high-risk population. The data collection period lasted 3 weeks beginning from 25 November 2022 to 16 December 2022. Prior to data collection, the research assistant was trained on the data collection tool, and this is where issues of patient confidentiality and data quality were emphasised. For the validity of the data collection tool, a pilot study was conducted on a sample of 34 patient files. The data file was password protected and the hard copies were kept in a locked cabinet accessible only to the researchers. The data were captured into an Excel spreadsheet, cleaned and coded before importation onto the Epi info seven statistical software package for the analyses. Descriptive statistics were used to describe the study sample. The mean and the standard deviation (s.d.) were used to present continuous variables such as age of the patients. Categorical data such as sex, were presented as proportions and percentages. Binary logistic analysis regression was conducted to determine the factors associated with unfavourable treatment outcomes. The results were presented as unadjusted odds ratios (uORs) with accompanying 95% confidence intervals (CI) and the *p*-values. All variables that yielded *p*-values ≤ 0.2 were included in a multivariable logistic regression analysis and the results were reported as adjusted odds ratios (aORs), with *p*-values < 0.05 indicating statistical significance.

### Ethical considerations

The study received ethical approval from the Sefako Makgatho Health Sciences University Research and Ethics Committee (SMUREC) (Ethical clearance number: SMUREC/H/39/2022:PG) and the Lesotho Ministry of Health Research and Ethics Committee. Approval to conduct the study was obtained from the district health management team (DHMT) and CHAL.

To ensure anonymity, the names of patients were not used during the data collection and analysis and files were coded for anonymity. Confidentiality was ensured by using a private room in the designated study sites when working with the patient files. Data were kept in the encrypted file on the researcher’s computer and hard copies under lock and key.

## Results

### Frequency distribution of sociodemographic characteristics of tuberculosis patients

Table 1 displays the sociodemographic characteristics of the 336 TB patients, of which 61% of patients were male. The mean age of patients was 44 years (s.d. = 16.9). Forty-six per cent were between the ages of 20 years and 40 years old, with the youngest and the oldest 7 years and 92 years old, respectively. Seventy-one per cent were unemployed, 88.7% resided in urban areas, with 33% who used substances.

**TABLE 1:** Sociodemographic characteristics of tuberculosis patients (*n* = 336).

Variable	Frequency (*n*)	Percentage (%)	Mean	s.d.
**Sex**				
Male	206	61.3	-	-
Female	130	38.7	-	-
**Age**	-	-	44	16.9
**Age group**				
≤ 10	2	0.6	-	-
> 10 – ≤ 20	14	4.2	-	-
> 20 – ≤ 30	67	20.0	-	-
> 30 – ≤ 40	84	25.5	-	-
> 40 – ≤ 50	54	16.1	-	-
> 50 – ≤ 60	47	14.0	-	-
> 60 – ≤ 70	36	11.0	-	-
> 70	32	9.5	-	-
**Residency**				
Urban	298	88.7	-	-
Rural	38	11.3	-	-
**Employment status**				
Employed	99	29.5	-	-
Unemployed	237	70.5	-	-
**Substance use**				
Yes	111	33.0	-	-
No	225	67.0	-	-

Out of the 220 patients who had weight and body mass index (BMI) recorded, the mean weight was 53.8 (s.d. = 11.8), with minimum and maximum weight of 16.5 kg and 105.8 kg, respectively. The mean BMI was 20.5 (s.d. 4.5) with minimum and maximum BMIs of 13 kg/m^2^ and 44 kg/m^2^, respectively.

### Frequency distribution of clinical characteristics of tuberculosis patients

Table 2 shows the clinical information of the study patients. It can be noticed that 89.6% of the patients were newly registered at the health facilities. Only 8.6% of patients had been moved in from another facility in the district. The remaining 1.8% comprised patients who had been moved in from health facilities located in another district within Lesotho or in another country. The key population profile indicates that 15.8% of patients were ex-mineworkers, with household members of the ex-miners, prison staff and prisoners constituting 5.8% of the key population.

**TABLE 2:** Clinical characteristics of the study patients (*N* = 336).

Characteristics	Frequency (*n*)	Percentage (%)
**Referral linkage**		
Moved in from another facility in the district	29	8.6
Newly registered in this facility	301	89.6
Transferred in from a facility in another district	3	0.9
Transferred in from outside Lesotho	3	0.9
**Key population profile**		
Ex-mine worker	53	15.8
Factory worker	42	12.5
Health worker	3	1.0
Household member of current miner	2	0.6
household member of ex- miner	10	2.9
Mine worker	5	1.5
None	211	62.8
Prisoner/prison staff	10	2.9
**Prior TB treatment history**		
Yes	47	14.0
No	289	86.0
**TB treatment supporter**		
Yes	316	94.1
No	20	5.9
**HIV status**		
Positive	189	56.2
Negative	147	43.8
**ART initiation (*n* = 189)**		
Yes	130	68.8
No	59	31.2
**Cotrimoxazole (*n* = 171)**		
Yes	151	88.3
No	20	11.8
**Other comorbid conditions (*n* = 336)**		
Yes	33	9.8
No	303	90.2
**Other comorbid conditions (*n* = 33)**		
Diabetes	4	12.1
Hypertension	15	45.5
Diabetes and hypertension	4	12.1
Immunosuppressants	3	9.1
Mental disorder	3	9.1
Other	4	12.1

TB, tuberculosis; HIV, human immunodeficiency virus; ART, antiretroviral therapy.

Ninety-four per cent of the patients had a treatment supporter. With regard to the HIV status, 189 were HIV positive, of which 68.8% were on antiretroviral therapy (ART). Out of the 189 HIV-positive patients, 90.5% had a record on cotrimoxazole, of which 88.3% being initiated. Of the 33 patients who had a comorbid condition, nearly half (45.5%) had hypertension, followed by 12.1% who had diabetes, and another 12.1% who had both diabetes and hypertension.

### Tuberculosis treatment outcomes

**FIGURE 1:**
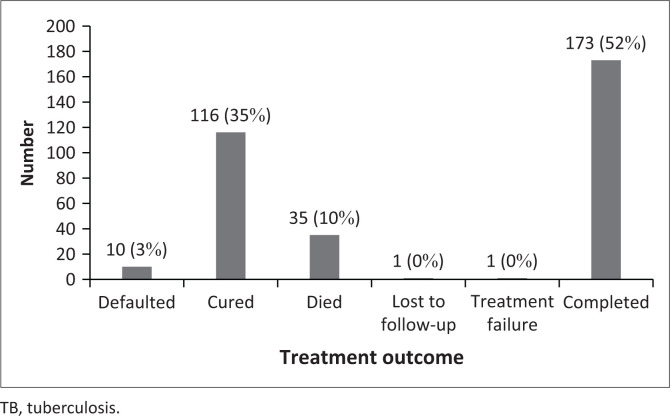
Tuberculosis treatment outcomes of tuberculosis patients (*N* = 336).

Figure 1 shows the treatment outcomes of the 336 TB patients at the end of the treatment phase. Fifty-two per cent of TB patients, completed treatment, 35% were cured, with 10% who died during the course of TB treatment, while 3% defaulted treatment.

### Factors associated with unfavourable treatment outcomes among tuberculosis patients

Table 3 shows the results from the binary and multivariable logistic regression analyses. The binary logistic regression analysis shows that the variables that had statistically significant associations with unfavourable treatment outcomes were the age of patient, having treatment supporter, HIV status and ART initiation status. Patients who were aged ≤ 44 years old were less likely to experience unfavourable treatment outcomes compared to those who were > 44 years old, uOR = 0.45 (95% CI: 0.24–085); *p* = 0.001. Those who had a treatment supporter were less likely to experience unfavourable treatment outcomes, compared to patients who did not have a treatment supporter, uOR = 0.21(95% CI: 0.08–0.55); *p* = 0.001. Patients who had a positive HIV status were more likely to experience unfavourable treatment outcomes compared to those who were HIV-negative, uOR = 2.00 (95% CI: 1.03–3.91), *p* = 0.04.

**TABLE 3:** Logistic regression analyses of the factors associated with tuberculosis treatment outcome (*N* = 336).

Variable	uOR	95% CI	*p*	aOR	95% CI	*p*
**Sex**						
Male	1.13	0.59–2.15	0.70	-	-	-
Female	Reference	Reference	Reference	Reference	Reference	Reference
**Age (years)**						
≤ 44	0.45	0.24–0.85	0.01*	0.39	0.19–0.78	0.007*
> 44	Reference	Reference	Reference	Reference	Reference	Reference
**Residency**						
Urban	0.85	0.33–2.16	0.73	-	-	-
Rural	Reference	Reference	Reference	Reference	Reference	Reference
**Employment status**						
Unemployed	1.26	0.62–2.53	0.52	-	-	-
Employed	Reference	Reference	Reference	Reference	Reference	Reference
**BMI (kg/m^2^)**						
≤ 20.5	1.22	0.47–3.16	0.68	-	-	-
> 20.5	Reference	Reference	Reference	Reference	Reference	Reference
**Treatment supporter**						
Yes	0.21	0.08–0.55	0.001*	0.22	0.08–0.62	0.004*
No	Reference	Reference	Reference	Reference	Reference	Reference
**HIV status**						
Positive	2.00	1.03–3.91	0.04*	3.34	0.92–12.15	0.07
Negative	Reference	Reference	Reference	Reference	Reference	Reference
**ART initiation**						
Yes	0.96	0.43–2.16	0.92	-	-	-
No	Reference	Reference	-	-	-	-
**Cotrimoxazole initiation**						
Yes	1.47	0.76–2.84	0.26	0.60	0.17–2.07	0.42
No	Reference	Reference	Reference	Reference	Reference	Reference
**Substance use**						
Yes	0.94	0.49–1.82	0.87	-	-	-
No	Reference	Reference	Reference	Reference	Reference	Reference
**Previous history of TB**						
Yes	0.92	0.38–2.19	0.85	-	-	-
No	Reference	Reference	Reference	Reference	Reference	Reference
**Comorbidity**						
Yes	2.17	0.91–5.14	0.08	1.62	0.64–4.14	0.31
No	Reference	Reference	Reference	Reference	Reference	Reference

95% CI, 95% confidence interval; uOR, unadjusted odds ratios; aOR, adjusted odds ratios; ART, antiretroviral therapy; BMI, body mass index; HIV, human immunodeficiency virus; TB, tuberculosis.

* , Statistically significant.

Multivariable analysis revealed that variables age of patient and having treatment supporter had persistent statistically significant associations with unfavourable treatment outcomes. Patients who were aged ≤ 44 years old were less likely to have unfavourable treatment outcomes compared to those who were aged > 44 years old, aOR = 0.39 (95% CI: 0.19); *p* = 0.007. Those who had a treatment supporter were less likely to have unfavourable treatment outcomes compared to those who had no treatment supporter, aOR = 0.22 (95% CI: 0.08–0.62); *p* = 0.004.

## Discussion

This study found 61% of the TB patients to be male. This corroborates the reports of TB being more common among male patients than females, and this is presumably because of social mixing patterns that drive male bias in TB, as well as the higher notification rates among the males than females.^19,20^ The finding of 70% unemployment among the TB patients in this study is higher than the national unemployment rate of Lesotho in 2022, estimated at 18%.^15^ Higher unemployment rates among TB patients compared to the general populations have been reported in other studies, including one from South Africa that found unemployment among TB patients at 54% compared to 30% national unemployment rate in 2022.^21^ Others have reported poor treatment outcomes including death and treatment interruption among unemployed TB patients compared to the employed TB patients.^22,23^

This study determined treatment success among the TB patients and found that 86% had been treated successfully. This figure does not only fall short of the end TB strategy target of 90% treatment success by 2025 but it also translates to 14% of unfavourable treatment outcomes. Treatment success rates reported from the SSA countries range between 45% in the Seychelles and 95% in Burundi.^15^ Unfavourable treatment outcomes observed in this study were attributed to 10% deaths that occurred in the course of treatment and 2.9% treatment default. The death of TB patients has been linked with delayed treatment initiation. Others have also reported of death between 7% and 28% TB patients during the course of treatment.^24,25,26^ This study found that 56% TB patients were co-infected with HIV. This is worrisome as individuals who have both TB and HIV have an acquired immunodeficiency syndrome (AIDS) defining condition. However, this finding is not surprising, as Lesotho has a high HIV and TB prevalence, with approximately 80% of HIV-infected patients said to have TB.^27^ Additionally, only 69% of those who were co-infected were on ART. This is worrisome as Lesotho is among the first countries that have adopted the ‘Test and Treat’ strategy in 2006.^28^ Early initiation of ART is known to reduce mortality among HIV-infected individuals with newly diagnosed TB.^29^

This study also investigated the factors that were associated with unfavourable TB treatment outcomes and found statistically significant lower odds among the patients who were ≤ 44 years old compared to those who were > 44 years old. Similarly, several studies have reported unfavourable treatment outcomes with advanced age, which is attributed to physiological deterioration and other comorbid conditions.^23,30,31^ Patients who had a treatment supporter had statistically significant lower odds of experiencing unfavourable treatment outcomes compared to those who did not have any treatment support. Similarly, others have reported higher odds of treatment success and low treatment interruption among TB patients who had treatment supporters than those who did not have any treatment support.^32^

## Conclusion

The death of patients while on TB treatment is unacceptable; these deaths could be averted through early diagnosis and prompt initiation on TB drugs. Addressing this as a national priority will also see improvement of the TSRs. A more in-depth study is recommended to be undertaken to thrash out the causes of death, time of death including age of patients at the time of death. Higher odds of unfavourable treatment outcomes among patients in the older age groups indicate increased vulnerability among older patients and calls for interventions that are targeted at improving the treatment outcomes among this vulnerable group. Lower odds of unfavourable treatment outcomes among HIV/TB co-infected patients on ART compared to their counterparts that were not on ART is an indication of how ART improves TB treatment outcomes among HIV-positive patients; hence early ART initiation should be encouraged for all TB patients who have a positive HIV test. The association between having treatment supporter and lowered likelihood of unfavourable treatment outcomes is a success story that needs to be reiterated among patients who are on long-term medication to improve adherence.
